# Comparison of intraocular pressure fluctuation and glaucoma progression rate between phakic and pseudophakic eyes in pseudoexfoliation glaucoma

**DOI:** 10.1038/s41598-023-49099-w

**Published:** 2024-01-02

**Authors:** Edward Kang, Ji-Hye Park, Chungkwon Yoo, Yong Yeon Kim

**Affiliations:** 1 Department of Ophthalmology, Korea University College of Medicine, Seoul, Korea; 2 Department of Ophthalmology, Korea University Ansan Hospital, 123 Jeokgeum-ro, Danwon-gu, Ansan-si, 15355 Gyeonggi-do Korea

**Keywords:** Outcomes research, Optic nerve diseases

## Abstract

The management of patients with concurrent pseudoexfoliation glaucoma (PXG) and cataract is challenging given its worse prognosis compared to other glaucoma types and the increased risk associated with cataract surgery. In this retrospective study, we investigated the long-term outcomes of cataract surgery in patients with PXG. We enrolled patients with PXG who had undergone cataract surgery at least 2 years previously and compared them with mean deviation (MD) matched patients with phakic eyes. The results showed that both groups experienced a decrease in MD, with the group of pseudophakic eyes exhibiting a significantly higher rate of decline (−2.15 ± 2.66 dB/year vs. −0.86 ± 0.95 dB/year; *P* = 0.040). Similarly, there was a trend towards more rapid thinning of the retinal nerve fiber layer in the pseudophakic group (−2.92 ± 2.34 μm/year vs. −1.79 ± 1.71 μm/year; *P* = 0.074). No significant differences in the intraocular pressure parameters were observed between the two groups. Multivariate analysis revealed that pseudophakic lens status was significantly associated with a faster rate of MD decline in patients with PXG (regression coefficient, −1.391; *P* = 0.022). These findings underscore the importance of close monitoring of patients with pseudophakic PXG to effectively manage glaucoma progression.

## Introduction

Glaucoma is a progressive optic neuropathy that causes structural and functional damage to the optic nerve head^1^. As increased age is an important risk factor for glaucoma development, many patients with glaucoma often have concurrent cataracts. Furthermore, long-term use of glaucoma medications and filtering surgery have been shown to accelerate the development of cataracts^2–4^. The effect of cataract surgery on glaucomatous eyes has been investigated. In terms of intraocular pressure (IOP) control, several studies have shown that cataract extraction provides a modest reduction in IOP and reduces dependency on glaucoma medications^5–7^. Moreover, a decrease in nocturnal IOP fluctuation was reported in patients with primary angle closure after cataract surgery^8^. However, there is conflicting evidence regarding the effect of cataract surgery on visual function. Hayashi et al. reported that cataract surgery could improve the mean deviation (MD) values in glaucomatous eyes, particularly when a central dense scotoma was present before surgery on two or fewer meridians^9^. Whereas other researchers found that all visual field (VF) indices indicated an accelerated decay rate after cataract surgery, suggesting worsening of visual function^10^.

Pseudoexfoliation (PEX) syndrome is an age-related systemic disorder characterized by abnormal elastosis and excessive accumulation of microfibrils at various sites in the body, including ocular tissues. The anterior segment of the eye is particularly affected, and accumulation of microfibrils poses a significant risk of developing pseudoexfoliation glaucoma (PXG). The accumulation of pseudoexfoliative material, injured trabecular meshwork (TM) endothelium, and deposits of released pigments may lead to IOP elevation. Moreover, the presence of pseudoexfoliative material is associated with disease progression in individuals who have already been diagnosed with glaucoma^11^. Based on the pathogenesis of PXG, some researchers have suggested washing out the pseudoexfoliative material located on the TM and iridocorneal angle as an treatment option^12–14^.

Previous studies have reported an etiological association between PEX and cataract^15,16^. Cataract surgery of the PXG eye is a challenging situation that can lead to several intraoperative and postoperative complications. A greater decrease in IOP was observed in the eyes with PEX than in those without PEX after cataract surgery^17,18^. Furthermore, Rao et al. reported a decrease in diurnal IOP fluctuations after cataract surgery in patients with PEX syndrome, regardless of angle occlusion^19^. In contrast, IOP spikes after phacoemulsification have been shown to occur more frequently in eyes with PEX^20,21^. Although there are studies reporting the effect of cataract surgery on IOP changes in patients with PXG, there is no study that compared the IOP fluctuation and the rate of glaucoma progression between phakic and pseudophakic eyes in patients with PXG. Because cataract surgery may widen the anterior chamber angle and wash out some pseudoexfoliative material, it may have a positive effect on the progression of glaucoma and fluctuations in IOP in individuals with PXG. Therefore, in this study, we compared IOP fluctuations and glaucoma progression rates in the phakic and pseudophakic eyes of patients with PXG. The findings of this investigation have the potential to enhance our understanding of PXG and assist in clinical decision making.

## Results

### Demographic and clinical characteristics

In this study, we reviewed the medical records of 98 patients with PXG who were followed up for more than 2 years, excluding 47 patients for the following reasons: previous history of glaucoma-related surgery or laser treatment (30 patients), advanced-stage glaucoma (11 patients), and history of other retinal diseases (6 patients). To compare the progression rate and IOP parameters, we matched the phakic and pseudophakic groups based on propensity scores, using global MD and baseline best-corrected visual acuity (BCVA) as the matching parameters. Of the 51 patients with PXG who met the inclusion criteria, 22 pairs of patients with phakic and pseudophakic eyes were selected.

Table 1 presents the participants’ demographic characteristics and baseline features. In the group with phakic eyes, the mean age was 73.91 ± 10.61 years old, and the mean follow-up period was 5.25 ± 3.91 years, whereas the group with pseudophakic eyes had a mean age of 75.18 ± 8.92 years old and a mean follow-up period of 5.85 ± 4.19 years. At baseline, the mean number of glaucoma medications for the group with pseudophakic eyes was 0.68 ± 0.78, which was significantly higher than that for the group with phakic eyes (0.18 ± 0.50, *P* = 0.016). However, at baseline, there was no significant difference in IOP between the two groups (*P* = 0.217). The anterior chamber depth (ACD) was significantly deeper in pseudophakic eyes (3.95 ± 0.60 mm) than in phakic eyes (3.04 ± 0.35 mm; *P* < 0.001). No significant differences at baseline were observed in the pattern standard deviation (PSD), MD, and retinal nerve fiber layer (RNFL) thickness between the two groups. (*P* = 0.071, *P* = 0.337, and *P* = 0.350, respectively).

**Table 1:** Demographic and clinical characteristics of participants.

	Phakic eye	Pseudophakic eye	*P* value*
Age (years)	73.91 ± 10.61	75.18 ± 8.92	0.669
Follow-up period (years)	5.25 ± 3.91	5.85 ± 4.19	0.615
Baseline medication	0.18 ± 0.50	0.68 ± 0.78	0.016
Baseline IOP (mmHg)	17.6 ± 3.7	15.9 ± 5.3	0.217^†^
BCVA	0.67 ± 0.15	0.70 ± 0.16	0.923
Spherical equivalent (D)	−0.12 ± 2.06	–0.39 ± 1.24	0.597
Axial length (mm)	23.59 ± 0.85	23.61 ± 1.19	0.955^†^
ACD (mm)	3.04 ± 0.35	3.95 ± 0.60	< 0.001^†^
MD (dB)	−4.59 ± 2.88	-6.18 ± 4.34	0.337
PSD (dB)	3.42 ± 2.87	5.32 ± 3.75	0.071
RNFL thickness (μm)	78.61 ± 12.40	74.14 ± 12.53	0.350^†^

*IOP* intraocular pressure, *BCVA* best-corrected visual acuity, *ACD* anterior chamber depth, *MD* mean deviation, *PSD* pattern standard deviation, *RNFL* retinal nerve fiber layer.

*Mann–Whitney *U* test.

^†^Independent *t*-test.

### IOP fluctuation and glaucoma progression rate

**Figure 1:**
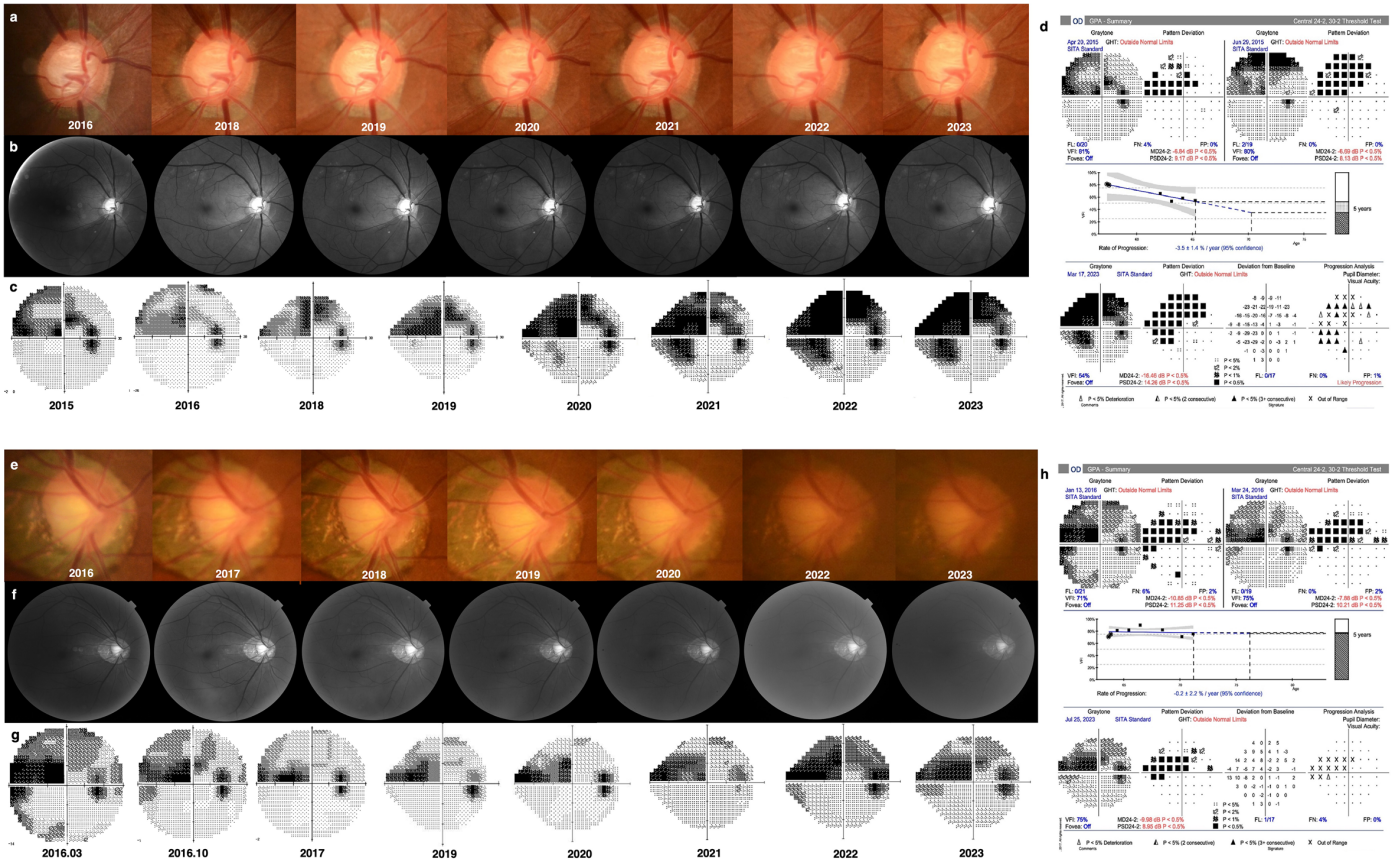
Representative cases of our study showing different visual field progression rate between (**a–d**) pseudophakic and (**e–h**) phakic eyes in patients with pseudoexfoliation glaucoma. (**a–d**) 65-year-old male had cataract surgery on 2012 and followed up for 8 years. His mean, peak, and standard deviation (SD) of intraocular pressure (IOP) were 14.2 mmHg, 21.0 mmHg, and 2.4 mmHg, respectively. The visual field index (VFI) progression rate was −3.5 ± 1.4%/year. (**e–h**) 63-year-old male visited the glaucoma clinic on 2016 and followed up for 7 years. During the follow-up period, his mean, peak, and SD of IOP were 15.4 mmHg, 23.0 mmHg, and 2.9 mmHg, respectively. His VFI progression rate was −0.2 ± 2.2%/year, much slower than in the pseudophakic eye.

During the follow-up period, glaucoma progression rate and IOP fluctuations were analyzed. A comparison between the two groups is presented in Table 2. At the final visit, the group with phakic eyes had a mean number of glaucoma medications of 1.41 ± 0.80, whereas the group with pseudophakic eyes had a mean number of 1.73 ± 1.12. Although the mean number of glaucoma medications was higher in the pseudophakic eye group, the difference between the two groups was not statistically significant. The peak IOP, mean IOP, and IOP standard deviation during the follow-up period were investigated in both groups, with the group with phakic eyes having values of 20.7 ± 5.5 mmHg, 15.4 ± 2.6 mmHg, and 2.57 ± 1.27 mmHg, respectively, and the group with pseudophakic eyes having values of 20.9 ± 7.8 mmHg, 14.9 ± 2.6 mmHg, and 2.91 ± 2.21 mmHg, respectively. In both groups, MD was found to be decreased, with the group with phakic eyes decreasing at a rate of −0.86 ± 0.95 dB/year and the group with pseudophakic eyes decreasing at a rate of −2.15 ± 2.66 dB/year. Furthermore, the rate of decrease was significantly higher in the group with pseudophakic eyes, indicating more rapid and pronounced visual field loss over time in this group than in the group with phakic eyes. (*P* = 0.040) During the follow-up period, the RNFL thickness decreased by −1.79 ± 1.71 μm/year in phakic eyes and by −2.92 ± 2.34 μm/year in pseudophakic eyes. Although the difference reached borderline significance, there was a trend towards more rapid thinning of the RNFL in the pseudophakic eye group (*P* = 0.074). Figure 1 shows the representative cases of phakic and pseudophakic eyes in patients with PXG with differing glaucoma progression rate.

**Table 2:** IOP fluctuation and glaucoma progression rate.

	Phakic eye	Pseudophakic eye	*P* value*
Baseline medication	0.18 ± 0.50	0.68 ± 0.78	0.016
Final medication	1.41 ± 0.80	1.73 ± 1.12	0.284
Baseline IOP (mmHg)	17.6 ± 3.7	15.9 ± 5.3	0.217^†^
Peak IOP (mmHg)	20.7 ± 5.5	20.9 ± 7.8	0.930
Mean IOP (mmHg)	15.4 ± 2.6	14.9 ± 2.6	0.480^†^
IOP standard deviation (mmHg)	2.57 ± 1.27	2.91 ± 2.21	0.533
MD slope (dB/year)	−0.86 ± 0.95	−2.15 ± 2.66	0.040
PSD slope (dB/year)	0.33 ± 0.72	0.45 ± 1.49	0.736
RNFL thickness slope (μm/year)	−1.79 ± 1.71	−2.92 ± 2.34	0.074

*IOP* intraocular pressure, *MD* mean deviation, *PSD* pattern standard deviation, *RNFL* retinal nerve fiber layer.

*Mann–Whitney *U* test.

^†^Independent *t*-test.

### Factors influencing the progression of MD and RNFL change rate

Table 3 presents the results of the univariate and multivariate analyses investigating the factors associated with the rate of MD progression. Univariate analysis revealed that having a pseudophakic eye (regression coefficient, −1.297; *P* = 0.037) and experiencing higher IOP fluctuations (regression coefficient, −0.378; *P* = 0.031) were significantly associated with worse outcomes. However, in the multivariate analysis, only lens status was associated with the MD rate (regression coefficient, −1.391; *P* = 0.022), indicating that patients with PXG and pseudophakia would have a faster MD decline.

**Table 3:** Variables associated with visual field progression rate.

MD rate	Univariate		Multivariate*	
	B	*P* value	B	*P* value
Age	−0.003	0.939		
Lens status (phakic eye referent)	−1.297	0.037	−1.391	0.022
ACD	−0.302	0.527		
Baseline MD	0.055	0.525		
Baseline RNFL thickness	−0.027	0.213		
Baseline IOP	−0.106	0.127	−0.106	0.119
Peak IOP	−0.042	0.378		
Mean IOP	−0.066	0.598		
IOP standard deviation	−0.378	0.031	−0.264	0.126

*IOP* intraocular pressure, *ACD* anterior chamber depth, *MD* mean deviation, *RNFL* retinal nerve fiber layer.

*Variables with a *P* < 0.20 in the univariate analysis were entered into the multivariate analysis.

When the factors associated with the rate of RNFL change were investigated, there was no significant association between the RNFL change rate and any of the variables investigated (Supplementary Table S1). Lens status showed a borderline association (regression coefficient −1.133, *P* = 0.074), whereas no significant association with RNFL change rate was found in the multivariate analysis (*P* = 0.794).

## Discussion

In this study, a significant association was found between cataract surgery and faster MD progression in patients with PXG. Patients who underwent cataract surgery showed a notably faster rate of MD progression than those who still had cataracts. We also observed a faster decrease in RNFL thickness in PXG with pseudophakic eyes compared with those with cataracts, which was a statistically borderline difference. Our multivariate analysis revealed that lens status was the only variable significantly associated with the MD progression rate in patients with PXG, while there was no significant association with IOP fluctuation or peak IOP. To the best of our knowledge, this study is the first longitudinal investigation of disease progression in patients with PXG based on lens status.

The specific pathogenic mechanisms underlying PXG are not completely understood. However, several studies have identified alterations in molecules such as vascular endothelial growth factor (VEGF), nitric oxide (NO), matrix metalloproteinases (MMP), transforming growth factor-beta (TGF-B), and lysyl oxidase-like protein (LOXL) in the aqueous humor. These molecular changes may be associated with accumulation of fibrillar material in the anterior segment^22–24^. Borazan et al. conducted a prospective study comparing VEGF levels in the aqueous humor among PXG, PEX syndrome, and control patients with cataract. They found that both the aqueous humor and plasma VEGF concentrations were higher in patients with PEX syndrome and PXG than in controls, suggesting a possible ischemic nature of this syndrome^23^.

PEX not only affects the re-architecture of the anterior segment but also has implications for the posterior segment, including the stiffness of the lamina cribrosa and peripapillary microvasculature. In our previous study, we observed decreased average peripapillary vessel density in the PXG compared to that in the primary open-angle glaucoma (POAG)^25^. Interestingly, there were no significant differences in lamina cribrosa depth (LCD) and anterior lamina cribrosa curvature depth (ALCD) between the two groups. Additionally, other researchers have reported thinning and alterations in mechanical strength in the lamina cribrosa of the PXG, rendering it more susceptible to deformation^26,27^. An atomic force microscopy imaging of the lamina cribrosa in PXG demonstrated a marked decrease in stiffness, indicating increased vulnerability to glaucomatous damage^28^. Based on the results of previous studies, it can be known that factors other than IOP play an important role in the disease progression of patients with PXG.

Multiple studies have shown that PEX makes cataract surgery more challenging^29,30^. However, the cataract surgery outcomes in PXG are still controversial. Jimenez-Roman et al. retrospectively investigated the postsurgical IOP and medication use in patients with PXG. They found that the PXG group exhibited a significant decrease of 20.39% in IOP and a substantial reduction of 34.46% in the number of medications used after a 12-month follow-up period^31^. Kristianslund et al. observed a significant decline in IOP by 2.6 ± 4.0 mmHg in the PEX group after an average follow-up period of 76 months following cataract surgery^32^. The IOP-lowering effect of cataract extraction may be influenced by several potential mechanisms. First, cataract surgery can increase aqueous outflow^33–35^. This can be achieved by widening the anterior chamber angle and deepening of postoperative ACD. In addition, increased prostaglandin production after surgery may further facilitate uveoscleral outflow. Second, cataract surgery may reduce aqueous production^36^. Researchers have noted that traction of the ciliary body caused by fibrosis and contraction of the posterior lens capsule can lead to a decrease in aqueous production. However, a recent study by Tekcan et al., reported a significant increase in the mean IOP and mean number of medications after uneventful cataract surgery in trabeculectomized PXG eyes^21^.

In general, cataract surgery has been thought to be beneficial in POAG and primary angle-closure glaucoma (PACG). Recent studies have evaluated the effect of early lens extraction in patients with PXG and reported that it is an effective and safe treatment option^37,38^. This may be attributed to factors such as widening of the anterior chamber angle and the potential washing effect after cataract surgery. However, the effect of cataract surgery on glaucoma progression in patients with PXG has not been definitively established. In a recent study conducted by Rodríguez-Una et al., the effect of early lensectomy on VF progression was compared between patients with symmetric and asymmetric PEX syndrome. The MD progression rate was –0.33 dB/year and −0.36 dB/year in patients with symmetric and asymmetric PEX syndrome, respectively, which did not differ significantly between the two groups^38^. Although the progression rates were similar, the authors suggested that early lensectomy may be more beneficial in patients with asymmetric PEX syndrome because these eyes showed a lower incidence of phacoemulsification-related complications.

In our study, although there were no differences in IOP fluctuations, the MD slope in patients with PXG with pseudophakia showed change at a faster rate than that in patients with PXG with cataracts. In our multivariate analysis, lens status was significantly associated with the rate of MD progression. Inconsistent with our findings, lens status was not a risk factor for glaucomatous progression in patients with PXG in a study by Moon et al.^39^. One potential reason for this difference could be that our study excluded patients who underwent glaucoma surgery, whereas their study included such patients. Consistent with our result, a previous study that compared POAG progression before and after cataract surgery reported a significant increase in glaucoma progression after cataract surgery^10^. However, in the subgroup analysis, there was no significant difference in the rate of glaucoma progression before and after cataract surgery in eyes that underwent trabeculectomy. It is possible that a fistula created by a previous trabeculectomy may attenuate the IOP spike during or early postoperative cataract surgery, consequently affecting the long-term progression rate of glaucoma.

There are several possible reasons for the faster progression of glaucoma in patients with PXG with pseudophakia. First, fluctuations in intraoperative IOP can cause glaucomatous damage^40^. IOP spikes were more common in PEX eyes compared to non-PEX eyes after cataract surgery^20^. Although we did not find a significant difference in IOP parameters between the two groups, there may have been an intraoperative IOP spike. Second, the vulnerable nature of the lamina cribrosa may contribute to accelerated axonal damage following cataract surgery. Widespread elastosis in the connective tissue of the lamina cribrosa contributes to glaucoma progression by interfering with its ability of the lamina cribrosa to adapt to IOP fluctuations. Further studies are necessary to provide additional evidence to support this association.

Our study had several limitations. First, it was a retrospective study, which may have introduced biases and limitations in data collection and analysis. In terms of IOP, we measured IOP during regular office hours, typically from 9 am to 5 pm, but did not measure IOP at the same time for all patients or consistently for a single patient. Therefore, the IOP standard deviation used in the study represents inter-visit IOP variation rather than diurnal variation. Second, the sample size was relatively small, which may limit the generalizability of our findings. Third, despite a faster rate of RNFL thinning in pseudophakic eyes compared to phakic eyes, the observed difference reached borderline significance (*P* = 0.074). The reasons for the inconsistent results between the rates of structural and functional progression remain unclear. However, this discrepancy may be attributed to the severity of glaucoma among study participants and the inherent limitations of the propensity score matching employed to align the two groups. Moreover, despite our exclusion of patients with dense cataracts, the media opacity in the phakic eyes may have obscured the genuine MD decline attributable to glaucoma, thereby potentially leading to a slower observed progression rate in these phakic eyes. Finally, due to the nature of our study design, we were unable to compare the rates of glaucoma progression before and after cataract surgery within the same individual. While it was not the primary focus of our study, we were able to observe changes in glaucoma progression rates before and after cataract surgery in patients with PXG (Supplementary Fig. S1). Future studies, particularly long-term prospective studies, are needed to overcome these limitations and provide more comprehensive insights into the impact of cataract surgery on glaucoma progression in patients with PXG.

In conclusion, we observed faster MD progression in patients with PXG who underwent cataract surgery than in those with MD-matched PXG patients with cataract. This indicates that patients with pseudophakic PXG may require more frequent follow-ups and aggressive IOP-lowering treatments.

## Methods

A retrospective review of medical records was conducted at the Korea University Ansan Hospital glaucoma clinic, including patients who visited the clinic between December 2008 and November 2022. This study was approved by the Institutional Review Board of Korea University Ansan Hospital (approval number: 2021AS0002) and the need for written informed consent was waived by our Review Board. This retrospective study was performed according to the tenets of the Declaration of Helsinki.

### Study population

Patients diagnosed with PXG with a minimum follow-up period of 2 years were included. In this study, glaucoma was diagnosed based on the presence of glaucomatous optic disc changes and reproducible glaucomatous VF defects. The following defects were identified as glaucomatous optic disc changes: (1) focal or diffuse neuroretinal rim thinning, (2) localized notching, or (3) RNFL defects. Glaucomatous VF defects were defined when two of the following three criteria were present: (1) a cluster of three or more non-edge contiguous points in the pattern deviation plot with a *P* value of < 1%, without crossing the horizontal meridian; (2) a pattern standard deviation (PSD) of < 5%; or (3) a glaucoma hemifield test result outside the normal limits. PXG was diagnosed based on the presence of pseudoexfoliative material observed on the anterior lens capsule, at the pupillary margin, or both, following mydriasis during slit lamp biomicroscopy examination.

The patients were divided into two groups: PXG with pseudophakic eyes and PXG with phakic eyes. Patients in the pseudophakic eye group underwent uncomplicated cataract surgery with intraocular lens implantation in a capsular bag at least two years prior to the study enrollment. Patients with complications, including intraoperative or postoperative events such as posterior capsular rupture, IOP spike, or endophthalmitis, were excluded from the study. Additionally, patients who underwent any form of glaucoma-related surgery or laser treatment, or had advanced glaucoma (MD < − 20 dB), or presented with cataracts graded ≥ 3 (nuclear color, nuclear opalescence, cortical, and posterior subcapsular cataract) according to the Lens Opacities Classification System (LOCS) III at baseline were excluded. If both eyes were eligible for inclusion, one eye was randomly selected from each patient for analysis. The pseudophakic and phakic eye groups were matched based on the propensity scores using global MD and BCVA as the matching parameters to compare glaucoma progression rates.

### Ophthalmic examinations

All patients received a comprehensive ophthalmic assessment, which included slit-lamp microscopy, BCVA measurement, refraction test, IOP measurement with Goldmann applanation tonometry, Humphrey 24-2 visual field testing, RNFL measurement with optical coherence tomography (OCT, Cirrus HD-OCT, Carl Zeiss Meditec, Inc., Dublin, CA, USA), central corneal thickness (CCT) measurement using specular microscopy (SP-2000P, Topcon, Tokyo, Japan), axial length and ACD measurements using IOLMaster (Carl Zeiss Meditec, Jena, Germany), and dilated 30° stereoscopic fundus photography and 50° red-free photography using a FF 450 plus IR camera (Carl Zeiss Meditec Inc., Dublin, CA, USA). Visual field testing was conducted using a Humphrey Field Analyzer (Carl Zeiss Meditec, Dublin, CA, USA). The 30-2 or 24-2 Swedish Interactive Threshold Algorithm (SITA) Standard program were used to test the visual field. For VF testing, the reliability indices were monitored for fixation losses, false-positive errors, and false-negative errors. Subjects' visual acuity was assessed using the Snellen chart. Patients with a BCVA value less than 0.5 were excluded from further analysis. Patients whose OCT images had poor-quality were excluded using the following criteria: (1) inadequate signal strength ≤ 5; (2) motion artifacts; (3) inadequate focus or poor clarity; or (4) segmentation failure.

Patients were followed up at 6-month intervals unless there was IOP elevation or suspicious glaucoma progression. Every 6 months, slit-lamp microscopy, IOP measurement, VF testing, and OCT examination were performed to monitor disease progression and assess the effectiveness of the treatment. For patients exhibiting progression, glaucoma medication was added, and re-examination was performed at a shorter follow-up interval. IOP parameters such as baseline IOP, peak IOP, mean IOP, and IOP standard deviation were evaluated during the follow-up period. The annual rates of change in VF parameters and RNFL thickness were calculated, and rates of change were compared between the two groups.

### Statistical analysis

Statistical analyses were performed using SPSS software version 21.0 (SPSS Inc., Chicago, IL, USA). The Mann–Whitney *U* test and independent *t*-test were used for nonparametric and normally distributed data, respectively. Propensity score matching was conducted to compare the progression rate and IOP parameters between the phakic and pseudophakic groups. The global MD and baseline BCVA values were covariates to calculate the propensity score, and the matching was processed using a greedy nearest neighbor. Univariate and multivariate linear mixed models were used to identify the potential risk factors associated with VF parameters and RNFL thickness change rates, including age, lens status, ACD, baseline MD, baseline RNFL thickness, baseline IOP, peak IOP, mean IOP, and IOP standard deviation. Variables with a *P* < 0.20 in the univariate analysis were entered into the multivariate analysis. A *P* value less than 0.05 was considered significant.

### Supplementary Information

Supplementary Information.

## Data Availability

The datasets generated during and/or analyzed during the current study are available from the corresponding author on reasonable request.
