# Supplementary material for: Comparison of intraocular pressure fluctuation and glaucoma progression rate between phakic and pseudophakic eyes in pseudoexfoliation glaucoma
Source: Sci Rep. 2024 Jan 2;14:6. doi: 10.1038/s41598-023-49099-w (PMC10761949; doi:10.1038/s41598-023-49099-w)

[Supplementary Information]

# Comparison of Intraocular Pressure Fluctuation and Glaucoma Progression Rate between Phakic and Pseudophakic Eyes in Pseudoexfoliation Glaucoma

Edward Kang, Ji-Hye Park*, Chungkwon Yoo, Yong Yeon Kim

Department of Ophthalmology, Korea University College of Medicine, Seoul, Korea

*Corresponding author: Ji-Hye Park, MD, PhD

Department of Ophthalmology, Korea University Ansan Hospital,

123 Jeokgeum-ro, Danwon-gu, Ansan-si, Gyeonggi-do 15355, Korea

E-mail: jennypark8321@gmail.com

Tel: 82-31-412-5160

FAX: 82-31-412-4267

**Supplementary Table S1. Variables Associated with Retinal Nerve Fiber Layer Thickness Change Rate**

| RNFL rate | Univariate | | Multivariate | |
|---|---|---|---|---|
| | **B** | ***P* Value** | **B** | ***P* Value** |
| Age | 0.004 | 0.904 | | |
| Lens status (phakic eye referent) | -1.133 | 0.074 | -0.261 | 0.794 |
| ACD | -0.694 | 0.147 | -0.650 | 0.374 |
| Baseline MD | -0.108 | 0.211 | | |
| Baseline RNFL thickness | -0.033 | 0.134 | -0.034 | 0.176 |
| Baseline IOP | 0.041 | 0.565 | | |
| Peak IOP | 0.000 | 0.992 | | |
| Mean IOP | 0.060 | 0.636 | | |
| IOP standard deviation | 0.015 | 0.934 | | |

*Variables with a $P < 0.20$ in the univariate analysis were entered into the multivariate analysis.

IOP, intraocular pressure; ACD, anterior chamber depth; MD, mean deviation; RNFL, retinal nerve fiber layer

**Supplementary Figure S1.** A case of 74-year-old female showing a different visual field (VF) progression rate before and after cataract surgery in a patient with pseudoexfoliation glaucoma.

A 74-year-old female underwent cataract surgery in her left eye without any complications at the age of 72 (in June 2021). (a) The disc photograph showed notching in the superotemporal and inferotemporal regions, which became more prominent in 2022. (b) The retinal nerve fiber layer (RNFL) thickness demonstrated predominant progression in the superotemporal and inferior regions in 2022 and 2023. (c) The visual field pattern deviation plot showed progression in the inferior hemifield, corresponding to the RNFL thinning. (d) The visual field index (VFI) plot showed a gradual decrease preoperatively, whereas a rapid decrease in VFI was observed after cataract surgery (red arrow). The baseline intraocular pressure (IOP) was 18.0 mmHg in the left eye, and no significant IOP elevation was observed during the follow-up period.

(MD, mean deviation)

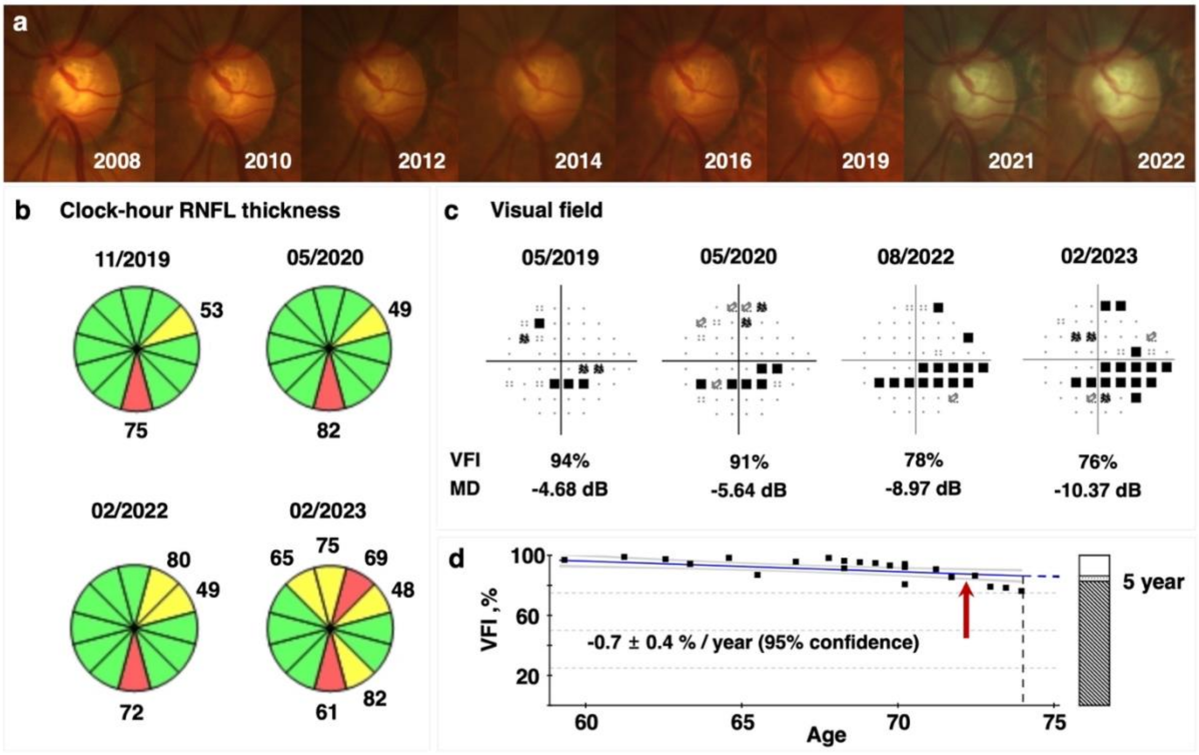

Supplement: Supplementary file 1 — Supplementary Information. [file 41598_2023_49099_MOESM1_ESM.pdf]
